# Periclase deforms more slowly than bridgmanite under mantle conditions

**DOI:** 10.1038/s41586-022-05410-9

**Published:** 2023-01-11

**Authors:** Patrick Cordier, Karine Gouriet, Timmo Weidner, James Van Orman, Olivier Castelnau, Jennifer M. Jackson, Philippe Carrez

**Affiliations:** 1 Univ. Lille, CNRS, INRAE, Centrale Lille, UMR 8207 - UMET - Unité Matériaux et Transformations, Lille, France; 2 Institut Universitaire de France, Paris, France; 3 Department of Earth, Environmental and Planetary Sciences, Case Western Reserve University, Cleveland, OH USA; 4 Laboratoire PIMM, Arts et Metiers Institute of Technology, CNRS, CNAM, HESAM University, Paris, France; 5 Seismological Laboratory, Division of Geological and Planetary Sciences, California Institute of Technology, Pasadena, CA USA

**Keywords:** Mineralogy, Geodynamics

## Abstract

Transport of heat from the interior of the Earth drives convection in the mantle, which involves the deformation of solid rocks over billions of years. The lower mantle of the Earth is mostly composed of iron-bearing bridgmanite MgSiO_3_ and approximately 25% volume periclase MgO (also with some iron). It is commonly accepted that ferropericlase is weaker than bridgmanite^1^. Considerable progress has been made in recent years to study assemblages representative of the lower mantle under the relevant pressure and temperature conditions^2,3^. However, the natural strain rates are 8 to 10 orders of magnitude lower than in the laboratory, and are still inaccessible to us. Once the deformation mechanisms of rocks and their constituent minerals have been identified, it is possible to overcome this limitation thanks to multiscale numerical modelling, and to determine rheological properties for inaccessible strain rates. In this work we use 2.5-dimensional dislocation dynamics to model the low-stress creep of MgO periclase at lower mantle pressures and temperatures. We show that periclase deforms very slowly under these conditions, in particular, much more slowly than bridgmanite deforming by pure climb creep. This is due to slow diffusion of oxygen in periclase under pressure. In the assemblage, this secondary phase hardly participates in the deformation, so that the rheology of the lower mantle is very well described by that of bridgmanite. Our results show that drastic changes in deformation mechanisms can occur as a function of the strain rate.

## Abstract (web summary)

The authors model the creep of MgO periclase at lower mantle pressures and temperatures, finding that it deforms more slowly than bridgmanite at mantle strain rate.

## Main

This work aims to model steady-state creep of periclase (MgO) under lower mantle conditions, with an emphasis on the role of extremely low strain rates. It is based on the extensive knowledge we have of the deformation of this oxide following (1) the large number of experimental studies carried out on this ceramic since the 1970s, including the more recent mineral physics studies taking into account the effect of pressure, and (2) the important modelling effort carried out in recent years. At room pressure, periclase is easily deformed by glide of $\frac{1}{2} < 110 >$ dislocations on {110} planes. The thermal activation of this mechanism can now be modelled from the atomic scale^4^, even at low strain rates^5^. Pressure strongly affects this mechanism and induces a transition of glide towards the {100} planes above 50 GPa^6–9^. At high temperature, dislocations glide without friction (athermal regime) and the flow stress results from dislocation interactions, leading, at large strains and strain rates, to a pronounced hardening^10^. At low strain rates, ionic diffusion can enhance recovery mechanisms and allow steady-state creep^11^. In this study, we model the influence of mantle pressures on steady-state creep of periclase by 2.5-dimensional (2.5D) dislocation dynamics (DD). We focus on four pressures: 30, 60, 90 and 120 GPa, which, along the geotherm^12^, correspond to 2,000, 2,300, 2,500 and 2,800 K respectively, at around depths of 800, 1,500, 2,100 and 2,700 km respectively.

## Diffusion coefficients

Dislocation climb in MgO is enabled by diffusion of Mg and O atoms, and the climb rate is controlled by the slower diffusing species. Diffusion in MgO has been studied for decades, and the mechanisms and rates of cation and anion diffusion are well established^13^. Mg and O diffuse using vacant sites on the cation and anion sublattices, respectively. Because the concentration of cation vacancies is much higher than that of anion vacancies, in both nominally pure synthetic and natural periclase samples, O diffusion is orders of magnitude slower than Mg diffusion, and is the rate-limiting step in dislocation climb. Whereas Mg diffusion is controlled by extrinsic vacancies, produced to charge-balance trivalent (and tetravalent) cation impurities, O diffusion is intrinsic, enabled by thermally produced Mg and O vacancy pairs (Schottky defects).

To calculate the oxygen self-diffusion coefficient, ${D}_{{\rm{Ox}}}^{{\rm{sd}}}$, over the full range of temperatures and pressures relevant to Earth’s mantle, we use the expression presented by Ita and Cohen^14^, which is derived from their theoretical results on vacancy formation and migration,

1 $${\rm{l}}{\rm{n}}{D}_{{\rm{O}}{\rm{x}}}^{{\rm{s}}{\rm{d}}}(P,T)={\rm{l}}{\rm{n}}({a}^{2}\,{\nu }_{{\rm{a}}})+\frac{{S}_{0}+P{S}_{0}^{{\prime} }}{{k}_{{\rm{B}}}}-\frac{{E}_{0}+P{V}_{0}+{P}^{2}{V}_{0}^{{\prime} }}{{k}_{{\rm{B}}}T}$$

where *P* and *T* are in GPa and K respectively, *a* is the lattice constant and *k*_B_ is the Boltzmann constant. The other parameters are listed in Table 1. The enthalpies are obtained by inverting the Arrhenius equation of diffusion coefficient:

2 $${D}_{{\rm{O}}{\rm{x}}}^{{\rm{s}}{\rm{d}}}{={D}}_{0}\exp \left(\frac{-\Delta {H}^{{\rm{s}}{\rm{d}}}}{{k}_{{\rm{B}}}T}\right)$$

where *D*_0_ = 7.2 × 10^−4^ m^2^ s^−1^ and Δ*H*^sd^ are, respectively, the exponential prefactor and the activation enthalpy^11^. The activation enthalpies for each set of pressure/temperature and the diffusion coefficients are given in Table 2.

**Table 1:** Oxygen self-diffusion parameters for MgO global fit as a function of pressure and temperature (from Ita and Cohen^14^)

Parameter	Symbol	Value
Attempt frequency	*ν*_a_	5.4 THz
Activation entropy at *P* = 0	*S*_0_	4 *k*_B_
*S*_0_ pressure derivative	*S’*_0_	0.02 *k*_B_ GPa^−1^
Activation energy	*E*_0_	0.4 × 10^−19^ J
Activation volume at *P* = 0	*V*_0_	16.7 Å^3^
*V*_0_ pressure derivative	*V’*_0_	−0.038 Å^3^ GPa^−1^

**Table 2:** Oxygen self-diffusion coefficients (m^2^ s^−1^) from equation (1) and activation enthalpy (eV) for MgO obtained from equation (2)

*P* (GPa)	*T* (K)	${{\bf{D}}}_{{\bf{Ox}}}^{{\bf{sd}}}$	*∆H*
30	2,000	6.61 × 10^−27^	9.15
60	2,300	3.05 × 10^−29^	11.59
90	2,500	3.46 × 10^−31^	13.56
120	2,800	5.26 × 10^−31^	15.09

**Fig. 1:**
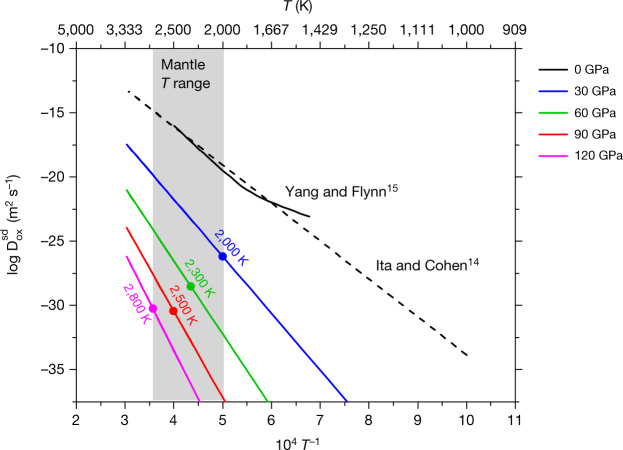
Oxygen diffusion coefficients in periclase. The solid black line represents the diffusion coefficients of Yang and Flynn^15^. The black dashed line corresponds to the diffusion coefficients of Ita and Cohen^14^ at 0 GPa. The other lines (blue, green, red and magenta) correspond to equation (1) at 30, 60, 90 and 120 GPa respectively. The range of temperatures considered in this study [2,000 K:2,800 K] is shown with the grey shading. The symbols (filled circles) correspond to the (*P*, *T*) conditions along the geotherm considered in the present study^12^ (Table 2). Source data

This expression produces values of ${D}_{{\rm{Ox}}}^{{\rm{sd}}}$ that are in agreement with the most reliable experimental results on intrinsic O diffusion from Yang and Flynn^15^ (Fig. 1). Higher values of ${D}_{{\rm{Ox}}}^{{\rm{sd}}}$ determined by Van Orman et al.^16^ at high pressure are considered to reflect the contribution of dislocations^17^ and/or other extended defects, as in most other studies of oxygen diffusion in MgO^13^. The results of Ita and Cohen^14^ are also similar to those presented in several later theoretical studies^18–20^.

## Modelling dislocation creep of periclase

**Fig. 2:**
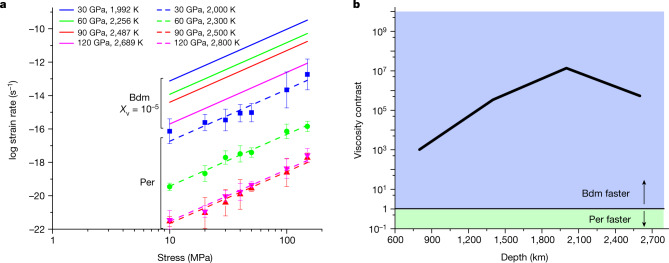
Creep rate modelling. **a**, Creep strain rates as a function of applied stress of bridgmanite (Bdm; solid line) and periclase (Per; symbols) at 30 GPa (blue), 60 GPa (green), 90 GPa (red) and 120 GPa (magenta). **b**, Viscosity contrast ${\eta }_{{\rm{Per}}}/{\eta }_{{\rm{Bdm}}}$ (calculated at 10 MPa) between the two pure phases, periclase and bridgmanite, as a function of depth. Source data

**Extended Data Fig. 1:**
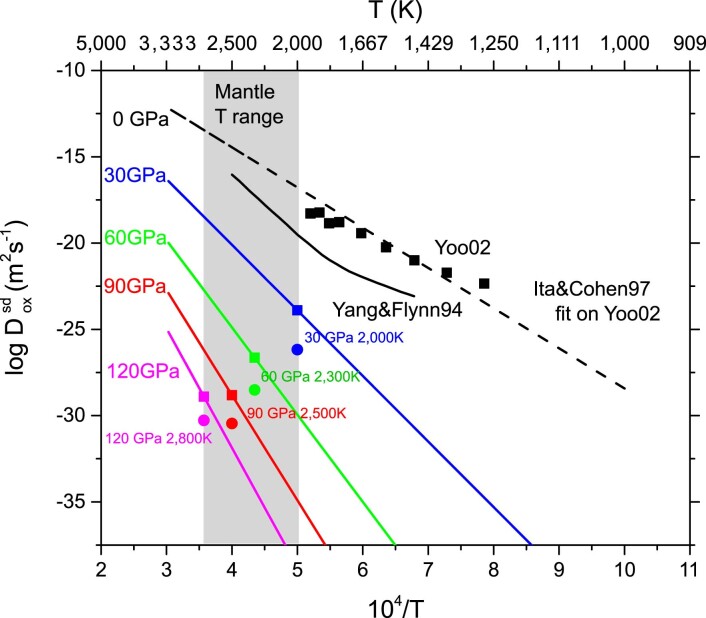
Oxygen diffusion coefficients in periclase. The black dotted line corresponds to the fit of Ita and Cohen^14^ diffusion law at 0 GPa on the experimental data of Yoo et al.^40^. Comparison with results of Yang and Flynn^15^ results is provided. The other lines (blue, green, red and magenta) correspond to this new set at 30, 60, 90 and 120 GPa respectively. The square symbols correspond to the diffusion coefficients along the geotherm deduced from this data. Comparison with data from Ita and Cohen^14^ used in the main text (filled circles symbols) is provided Source data

**Extended Data Fig. 2:**
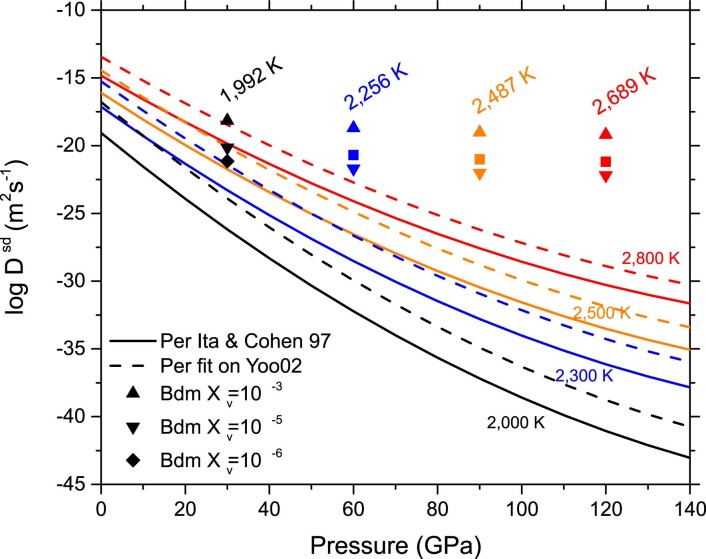
Pressure dependence of ionic diffusion. The lines represent oxygen diffusion coefficients in periclase (Per). We compare the data of Ita and Cohen^14^ (solid lines) to those obtained from the pressure dependence of Ita and Cohen^14^ but anchored on data from Yoo et al.^40^ (dotted lines). The symbols correspond to magnesium diffusion in bridgmanite (Bdm) considering a range of vacancy concentrations from 10^−3^ to 10^−6^ Source data

**Extended Data Fig. 3:**
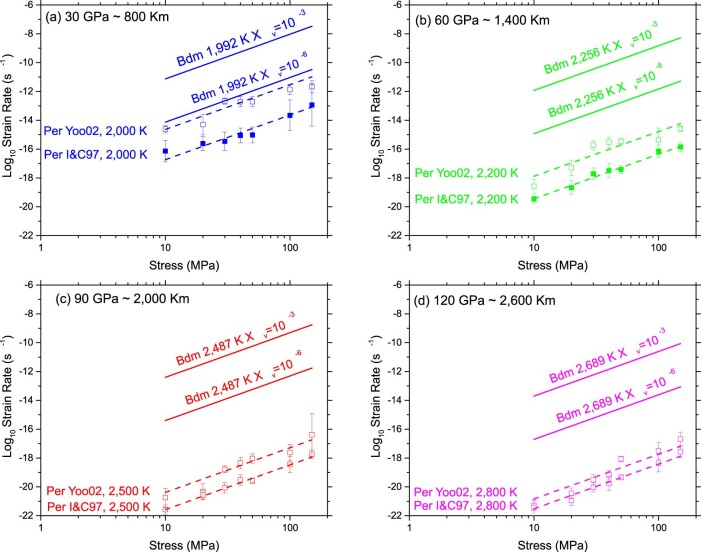
Creep laws in bridgmanite (Bdm) and periclase (Per). In this figure we illustrate the influence of possible uncertainties on the diffusion coefficients in bridgmanite and periclase. For bridgmanite (Bdm) vacancy concentrations of 10^−3^ and 10^−6^ are considered. Creep in periclase is based on diffusion data of Ita and Cohen^14^ and Yoo et al.^40^. Whatever the set of data considered, bridgmanite always deform faster than periclase Source data

**Extended Data Fig. 4:**
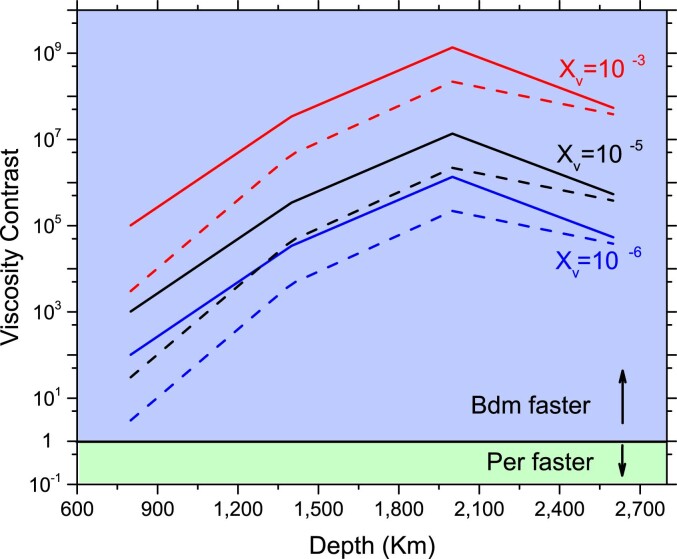
Viscosity contrast between bridgmanite (Bdm) and periclase (Per). In this figure we illustrate the influence of possible uncertainties on the diffusion coefficients in bridgmanite and periclase. For bridgmanite (Bdm) vacancy concentrations in the range 10^−3^ to 10^−6^ are considered. Creep in periclase is based on diffusion data of Ita and Cohen^14^ (Solid lines) and Yoo et al.^40^ (dotted lines). Whatever the set of data considered, bridgmanite always deform faster than periclase Source data

To model plasticity of periclase under mantle conditions we use a 2.5D DD simulation approach already benchmarked for periclase at ambient pressure^11^. DD simulation is a modelling tool that describes the collective motion and interactions of dislocations at the mesoscale. This technique is based on continuum elasticity theory, which provides the description of the elastic field induced by dislocations in a crystal, their interactions with each other and with respect to the stress field resulting from external loading. Within our DD code, dislocation glide is coupled with climb to investigate high-temperature creep (Methods). Our creep results are presented in Fig. 2a, which shows the creep strain rates obtained from the 2.5D DD simulations as a function of the creep stress for the four (*P*, *T*) conditions selected. A constant slope is found for all these strain rate–stress curves, which gives the value of the power law stress exponent, *n* = 3.1. A strong pressure dependence is found between (30 GPa, 2,000 K) and (90 GPa, 2,500 K), whereas creep rates at (90 GPa, 2,500 K) and (120 GPa, 2,800 K) are very close due to very similar values for the diffusion coefficients (Fig. 1 and Table 2). On Fig. 2a we compare the creep rates of periclase with those of bridgmanite at the same depths. For bridgmanite, we use the results of Reali et al.^21^ for pure climb creep (PCC) with a vacancy concentration *X*_v _= 1 × 10^−5^; these results were shown to be in good agreement with empirical geophysical constraints on the viscosity profile of Earth’s lower mantle. Bridgmanite and periclase exhibit similar stress exponents. However, we see that these calculations lead to bridgmanite having higher creep rates than periclase at any depth. To better illustrate this difference, we report, on Fig. 2b, the viscosity contrast between those two phases, taken as the ratio ${\eta }_{{\rm{Per}}}/{\eta }_{{\rm{Bdm}}}$. We see that under lower mantle conditions bridgmanite creep rates are 10^3^ to 10^7^ greater than periclase creep rates. This result reflects the fact that in the lower mantle, anionic diffusion in periclase is much slower than cationic diffusion in bridgmanite (Extended Data Fig. 2). In the online Methods section and Extended Data Figs. 1–4 we consider other diffusion data from the literature for periclase and other vacancy concentrations in bridgmanite and show that the conclusions stated above remain valid in any case.

## Laboratory versus nature

**Fig. 3:**
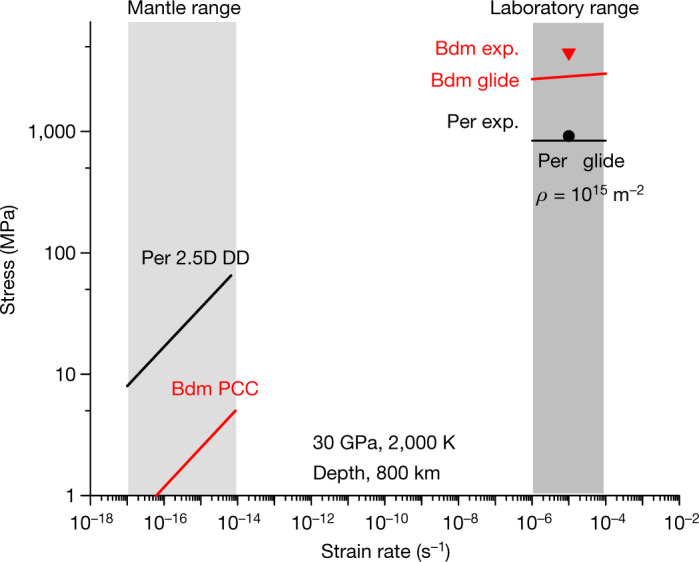
Influence of strain rate on deformation mechanisms. This figure corresponds to approximately 30 GPa, 2,000 K, where the experimental (exp.) data of Girard et al.^3^ (symbols) can be compared to numerical models (lines). At laboratory stresses and strain rates numerical models based on dislocation glide are in fair agreement with experimental data and show that bridgmanite (Bdm) is stronger than periclase (Per). At mantle strain rates, creep (Weertman creep modelled by 2.5D DD in periclase (see text) and pure climb creep (PCC) in bridgmanite) is controlled by ionic diffusion, which leads to an inversion of rheology, with periclase deforming more slowly than bridgmanite. Source data

In this paragraph we address the influence of strain rate on deformation mechanisms. Our calculations showing that bridgmanite deforms faster than periclase under mantle conditions seem to contradict the common intuition that the silicate is stronger than the oxide. Beyond general considerations^1^ or studies of analogous compounds^22,23^, Girard et al.^3^ conducted experiments under mantle conditions of pressure and temperature that show very clearly that ferropericlase accommodates most of the strain compared to bridgmanite. We show here that there is no fundamental opposition between modelling and experiments and that our models are able to account equally for natural and experimental conditions. The experiments of Girard et al.^3^ are conducted at a strain rate of 3 × 10^−5 ^s^−1^ and under very high stresses. Under these laboratory conditions, dislocation glide is expected as the dominant deformation mode. For bridgmanite, dislocation glide is thermally activated under these conditions and a model based on the Orowan equation can be built^24^. The stress values predicted by this model are reported on Fig. 3. They are close to the experimental values (slightly lower as this model was constructed for single crystals and does not involve grain boundaries). At 30 GPa and 2,000 K, dislocations in periclase glide in the athermal regime^6^. Several deformation experiments of periclase report that in the athermal regime, no steady state is reached and that strain hardening occurs^25–27^ under the influence of dislocation–dislocation interactions. The origin of this strain hardening has been studied and modelled by Amodeo et al.^10^. It is well described by the Taylor mechanism, and the athermal stress *τ*_*f*_ required to overcome the forest dislocation network is given by:

3 $${\tau }_{f}=\mu b\frac{{\rm{ln}}\left(1/b\sqrt{\beta {\rho }_{{\rm{f}}}}\right)}{{\rm{ln}}\left(1/b\sqrt{\beta {\rho }_{{\rm{ref}}}}\right)}\sqrt{\beta {\rho }_{{\rm{f}}}}$$

where *μ* is the shear modulus, *b* the modulus of the Burgers vector, *β* is a forest strengthening coefficient calibrated by Amodeo et al.^10^, ${\rho }_{{\rm{f}}}$ is the forest dislocation density and ${\rho }_{{\rm{ref}}}$ is the reference dislocation density. Transmission electron microscopy observations^28^ on the specimens of Girard et al.^3^ confirm this view and show a very large dislocation density estimated at over 10^15 ^m^−2^. Using this dislocation density and equation (3) leads to an excellent description of experimental values for the deformation of ferropericlase under shallow lower mantle *P*, *T* conditions at laboratory strain rates (Fig. 3). Our modelling approach allows us to reproduce very well the experimental data of Girard et al.^3^ and confirm that, under laboratory conditions, bridgmanite is indeed expected to be stronger than ferropericlase. It should be noted that the flow stress value for ferropericlase corresponds to a very high hardening and that under more ‘gentle’ laboratory deformation conditions (that is, smaller strains or more recovery), the rheology contrast under laboratory conditions should be even larger. At 30 GPa, oxygen diffusion is already slow, and strain rates much lower than those obtainable in laboratory experiments would be required for climb to become active and promote the recovery necessary for steady-state creep.

## Deformation of a bridgmanite-periclase aggregate

Our model places first-order constraints on the intrinsic properties of the major phases of the lower mantle. The rheological behaviour of rocks in the lower mantle certainly involves more complexity. A first step towards this direction is to use dedicated micromechanical models, to connect the viscoplastic behaviour of a polycrystalline aggregate representative of a lower mantle assemblage with those of the individual phases, bridgmanite and periclase, and, at the same time, quantify the contribution of both phases to the effective strain rate, in relation with the aggregate microstructure.

**Fig. 4:**
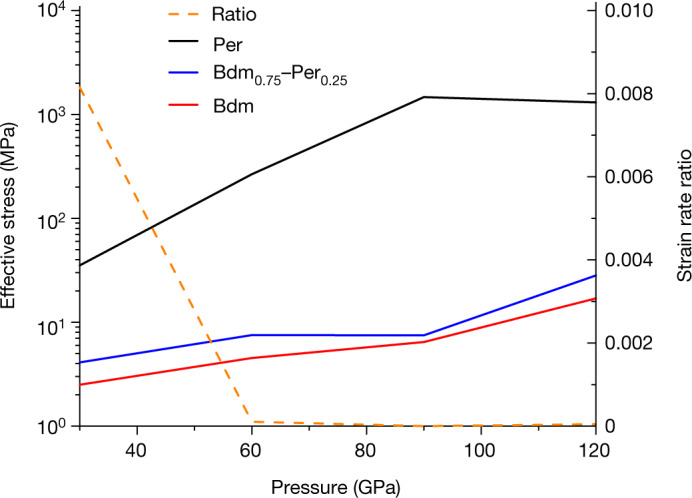
Rheology of bridgmanite–periclase aggregates. Pressure dependent flow stress ${\widetilde{\sigma }}_{0}$ for a bridgmanite (Bdm)–periclase (Per) aggregate deformed in creep at 10^−15 ^s^−1^. Calculations are performed within the POSO-SC scheme, with a volume fraction of periclase of 0.25 and a vacancy concentration in bridgmanite of *X*_v_ = 1 × 10^−5^. The ratio of strain rate (periclase/bridgmanite) is also indicated. Flow stresses *σ*_0_ of pure bridgmanite (in red) and pure periclase (in black) are also shown for comparison. Source data

**Extended Data Fig. 5:**
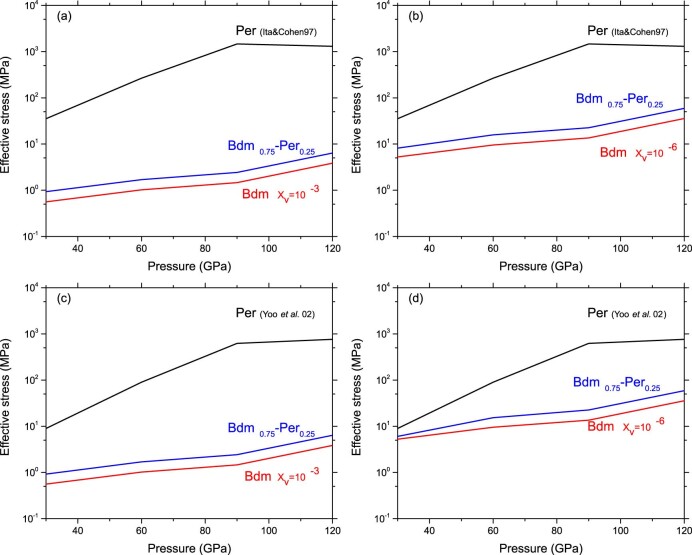
Pressure dependence of the aggregate rheology. The flow stress ${\widetilde{\sigma }}_{0}$ (blue line) for a bridgmanite (Bdm) – periclase (Per) aggregate (volume fraction of MgO: 0.25), is obtained with the POSO Self-Consistent scheme, for vacancy concentrations of 10^−3^ and 10^−6^ in bridgmanite and two oxygen diffusion coefficients for periclase (from Ita and Cohen^14^ and Yoo et al.^40^). Flow stresses of pure bridgmanite (red line) and pure periclase (black line) are also shown for comparison Source data

We have therefore applied a state-of-the-art scale transition homogenization method^29^ (partially optimized second-order self-consistent procedure, denoted POSO-SC) adapted for multiphase aggregates with nonlinear rheologies, such as those considered here. The model considers an isotropic microstructure, that is, a random mixture of bridgmanite and periclase with no preferred elongated shape nor crystallographic texture for each phase. Details are given in the Methods. The effective viscoplastic behaviour can be expressed with a nonlinear creep law with a stress sensitivity *n* = 3.1 (see Methods). Figure 4 shows the results obtained for a vacancy concentration *X*_v_ = 1 × 10^−5^ in bridgmanite and a volume fraction of periclase of 25% (alternative figures considering different combinations of diffusion coefficients are presented in Extended Data Fig. 5). For pressures in the range 30–120 GPa, the effective rheology (blue line) lies very close to that of pure bridgmanite (red line), with the flow stress values differing by a factor of only around 1.65, whereas the flow stress of pure periclase (black line) is one to two orders of magnitude larger. Periclase deforms at least 100 times slower than bridgmanite in the aggregate and its contribution to the effective strain rate is negligible. In the online Methods section, we show that the volume fraction of periclase would have to exceed 40% to significantly affect the rheology of the assemblage. The presence of iron in MgO may affect anionic diffusivity but does not change our conclusions for the bulk lower mantle (see online Methods section).

Following the widespread acceptance that ferropericlase is the weakest phase^1^, and inspired by the experimental results of Girard et al.^3^, several studies^30–32^ have discussed the implication of the morphology of ferropericlase (either forming a load-bearing framework or an interconnected weak layer^33^) on the rheology of the lower mantle. Our study shows that under the conditions (mainly strain rate) of the lower mantle, the behaviour of periclase is radically different and that this fundamental assumption is now challenged. Periclase, minor in volume, hardly deforms and probably induces little evolution of the microstructure. Consequently, the rheology of the lower mantle is very well described by that of bridgmanite deforming by pure climb creep.

## Methods

### Dislocation dynamics calculations

The 2.5D DD simulation method is used throughout this study to model the creep properties of periclase under lower mantle conditions under a constant creep stress ranging from 10 to 150 MPa. We consider the *P*, *T* conditions on the geotherm of Stacey and Davis^12^ at 30, 60, 90 and 120 GPa, and use the periclase elastic properties from Karki et al.^34^ (Extended Data Table 2).

To capture the statistically representative volume element of the microstructure, dislocations of Burgers vector **b** = $\frac{1}{2}$<110> are introduced edge-on in two intersecting slip systems within a square element of typical cell size *L*_*x*_ *=* *L*_*y*_ between 8 to 60 µm (function of the desired dislocation densities) and periodic boundary conditions are applied. As extensively described in some of our previous works^11^, specific characteristics of a realistic MgO microstructure are introduced as local rules, including junction formation strengthening and a dislocation multiplication rate to allow random introduction of fresh dislocations of opposite signs on all slip systems. All local rules are parameterized according to experimental observations or three-dimensional DD simulations as described in Reali et al.^11^.

For a realistic simulation of the creep behaviour, we use a combination of glide and climb events to control the displacement of dislocations in the simulation volume. The Burgers vector contained in the reference plane defines the slip direction, whereas the climb direction is defined as the one in the reference plane, orthogonal to the Burgers vector. The positive orientation of the climb direction is taken along the vacancy emission direction. During a given timestep of the simulation, dislocations are displaced along the glide or climb direction according to the velocity formulation presented below.

In our simulations, dislocations glide in the athermal regime according to a « free-flight » velocity *v*_*g*_, which is a function of the effective resolved shear stress *τ* *=* *τ*_app _*+* *τ*_int_ (that is, including the applied resolved shear stress and the elastic interaction stress):

$${v}_{{\rm{g}}}=\frac{b\tau }{B}$$

As, in the range of *P* and *T* conditions investigated here, the lattice friction of MgO vanishes^5,6^, the glide velocity is thus expressed with a viscous drag coefficient *B*. Such a coefficient is known to be temperature and material dependent. Thus, we consider a linear scaling of *B* with temperature^35^ and use as a starting point the experimental value recorded at 300 K^36^.

Climb events occur through the self-diffusion of vacancies adsorbed or emitted by the dislocation line. To describe the climb rate in steady-state creep equilibrium conditions, we use a dislocation climb velocity *v*_c_ expressed as follows^35^:

$${v}_{{\rm{c}}}=\alpha \frac{{D}^{{\rm{sd}}}}{b}\left[\exp \left(\frac{{\tau }_{{\rm{c}}}\,\varOmega }{{k}_{{\rm{B}}}T}\right)-\frac{{c}_{{\rm{\infty }}}}{{c}_{0}}\right]$$

where *τ*_c_ is the climb stress, calculated similarly to *τ* but resolved here along the climb direction. $\alpha =2\pi /{\rm{ln}}(R/{r}_{{\rm{c}}})$ is a geometrical factor that describes the cylindrical geometry of the vacancy flux field around the dislocation line, where *R* and ${r}_{{\rm{c}}}$ represent the two radii of the cylindrical surfaces through which the vacancy flux is calculated. Again, in the range of temperature investigated, the dislocation line must contain a high density of jogs, and thus be saturated with jogs, enabling vacancies to be absorbed or emitted instantaneously by the dislocation. Here, ${r}_{{\rm{c}}}$ is the dislocation core capture radius and *R* is taken as a fraction of the average dislocation distance. Being within the logarithmic term, the $R/{r}_{{\rm{c}}}$ ratio does not significantly affect the climb velocity values and here is taken as constant and equal to 100^37^. *Ω* is the formation volume of vacancies in the Mg and O sites and is calculated from the unit cell volume of MgO—that is, $\varOmega ={a}^{3}/Z$ where *Z* = 4 is the number of formula units per unit cell. *c*_∞_ and *c*_0_ are the vacancy concentrations far from the dislocation and the equilibrium vacancy concentration in the bulk volume, respectively. Following Boioli et al.^37^, we assume that far away from the dislocations (that is, on the external limit *R* of the cylindrical flux) the vacancy concentration is constant and equal to the equilibrium concentration in the bulk volume (*c*_∞ _= c_0_). Finally, *D*_sd_ is the vacancy self-diffusion coefficient, which controls the flow of atomic species from and to the climbing dislocation.

### Micromechanical model of the rheology of the aggregate

#### Local behaviour

We apply state-of-the-art formulations of nonlinear homogenization methods to estimate and bound the effective viscosity of a two-phase material constituted by bridgmanite and periclase with various volume fractions, at pressures and temperatures relevant for the lowermost part of Earth’s mantle. These phases are supposed to be randomly mixed so that the effective mechanical behaviour of the whole aggregate can be considered isotropic. Bridgmanite and periclase are considered to be two isotropic viscous phases too. In reality, they are both made of grains, but we consider that these grains are equiaxed and randomly oriented so that, on average, the polycrystalline aggregates can be replaced by two homogenous and isotropic phases. The role of grain or phase boundaries is not explicitly taken into account. We are interested in the effective rheological behaviour in the permanent (that is, secondary) creep regime. Thus, the elastic behaviour of both phases does not come into play. At the scale of each phase, the viscoplastic behaviour can be described by the following nonlinear creep law:

$${\dot{\varepsilon }}_{{\rm{eq}}}={\dot{\varepsilon }}_{0}{\left(\frac{{\sigma }_{{\rm{eq}}}}{{\sigma }_{0}}\right)}^{n}$$

with ${\dot{\varepsilon }}_{0}$ a reference strain rate, *σ*_0_ a reference stress, *n* the stress sensitivity, ${\dot{\varepsilon }}_{{\rm{eq}}}$ the equivalent strain rate and *σ*_eq_ the equivalent stress, defined as:

$${\dot{{\varepsilon }}}_{{\rm{e}}{\rm{q}}}=\sqrt{\frac{2}{3}{\dot{{\varepsilon }}}_{ij}{\dot{{\varepsilon }}}_{ij}},{\dot{{\sigma }}}_{{\rm{e}}{\rm{q}}}=\sqrt{\frac{3}{2}{{\sigma }{\prime} }_{ij}{{\sigma }{\prime} }_{ij}}$$

with summation over repeated indices (Einstein convention), ${\dot{\varepsilon }}_{{ij}}$ and ${\sigma {\prime} }_{{ij}}$ components of the strain rate tensor and deviatoric stress tensor, respectively. We chose here to take ${\dot{\varepsilon }}_{0}=1\times {10}^{-15}{{\rm{s}}}^{-1}$, that is, a value typical for lower mantle convection. Doing so, the *σ*_0_ values are of the order of magnitude of the equivalent stress encountered in situ during mantle flow. Values of *σ*_0_ for bridgmanite and periclase are fitted from the creep laws of Fig. 2 and available from the data deposit. The stress sensitivity *n* is the same for both phases, *n* = 3.1.

#### Effective behaviour

The effective behaviour reads in a similar way as the local behaviour:

$${\dot{\bar{\varepsilon }}}_{{\rm{eq}}}={\dot{\varepsilon }}_{0}{\left(\frac{{\bar{\sigma }}_{{\rm{eq}}}}{{\widetilde{\sigma }}_{0}}\right)}^{\widetilde{n}}$$

where $\dot{\bar{\varepsilon }}$ and $\bar{\sigma }$ are the effective strain rate and stress tensors, respectively, given by the volume average (denoted $\left\langle \bullet \right\rangle$) of local strain rate and stress fields: $\dot{\bar{\varepsilon }}=\left\langle \dot{\varepsilon }\right\rangle$ and $\bar{\sigma }=\left\langle \sigma \right\rangle$. $\widetilde{n}$ is the effective stress sensitivity, which can, in general, be inferred from the homogenization procedure. In the case considered here, things are simpler as bridgmanite and periclase exhibit the same *n* value. Therefore $\widetilde{n}=n=3.1$. Finally, ${\widetilde{\sigma }}_{0}$ is the effective flow stress associated with the (nonlinear) viscosity of the aggregate in situ. It is determined by the homogenization procedure detailed below.

#### Homogenization procedure

To describe the possible rheology of an assemblage representative of the lower mantle, one can reasonably assume that bridgmanite and periclase phases are randomly mixed. In that case, a good estimation of the effective behaviour is provided by the Self-Consistent scheme (denoted SC below), based on a fully disordered microstructure^38,39^. To extend the SC scheme to nonlinear rheologies, as here, we have made use of the very efficient and accurate linearization procedure (so-called partially optimized second-order procedure (POSO)) of Ponte Castaneda^29^.

### Sensitivity to calculation parameters

The calculations presented in the main text are made based on the diffusion coefficients for pure MgO periclase and for MgSiO_3_ bridgmanite with a vacancy concentration of 10^−5^. For the calculation of the aggregate rheology, we considered a periclase proportion of 25% by volume. In the following, reasonable alternatives to these choices are discussed to assess the robustness of our conclusions.

#### Diffusion coefficients

**Extended Data Fig. 6:**
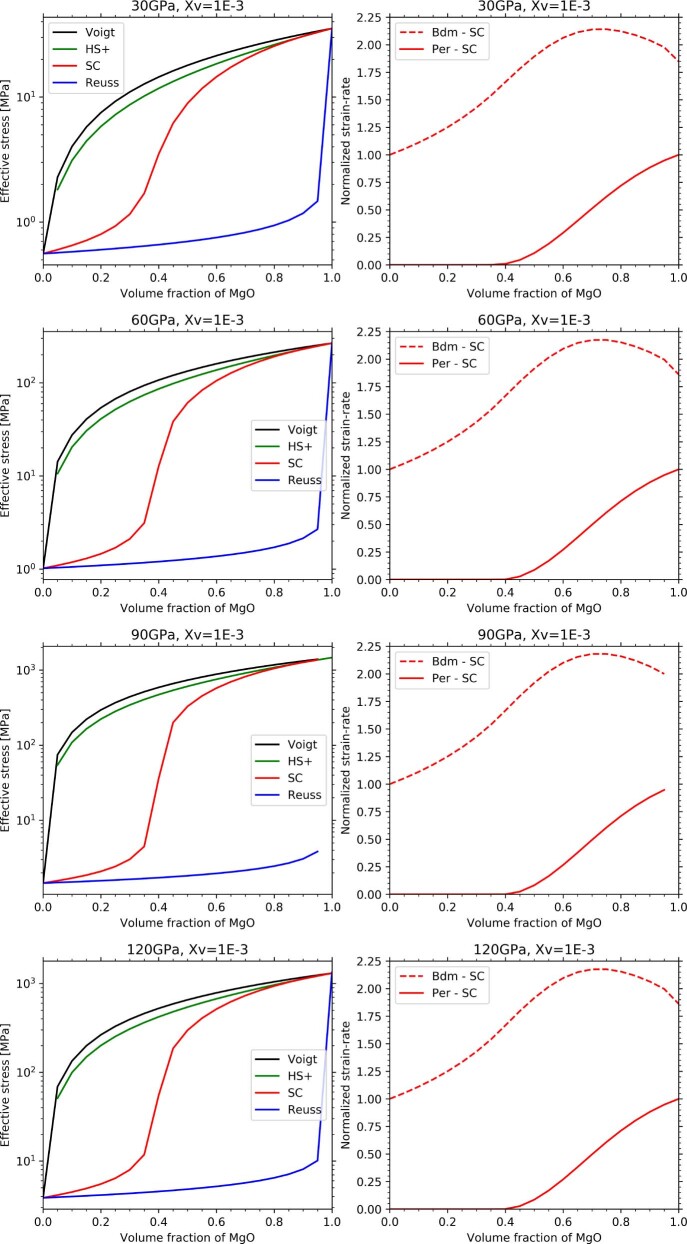
Influence of periclase volume fraction (*X*_*v*_ = 10^−3^). Behavior for a vacancy concentration in bridgmanite of *X*_*v*_ = 10^−3^ and oxygen diffusion coefficient in periclase from Ita & Cohen^14^ at various pressures. Left: Effective flow stress ${\widetilde{\sigma }}_{0}$ based on four homogenization procedures: Reuss, Voigt, Hashin-Strikman (HS+) and the POSO extension of the Self-Consistent scheme (SC). Right: Relative strain-rates in bridgmanite and periclase. Source data

**Extended Data Fig. 7:**
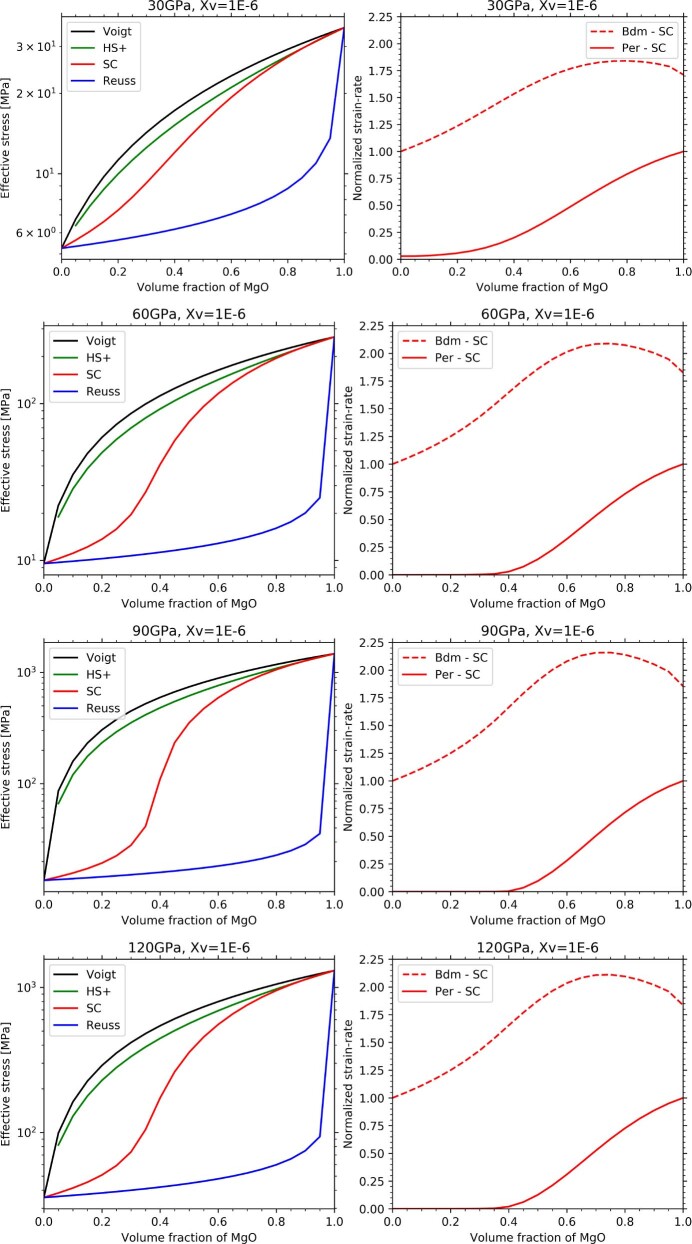
Influence of periclase volume fraction (*X*_*v*_ = 10^−6^). Behavior for a vacancy concentration in bridgmanite of *X*_*v*_ = 10^−6^ and oxygen diffusion coefficient in periclase from Ita & Cohen^14^ at various pressures. Left: Effective flow stress ${\widetilde{\sigma }}_{0}$ based on four homogenization procedures: Reuss, Voigt, Hashin-Strikman (HS+) and the POSO extension of the Self-Consistent scheme (SC). Right: Relative strain-rates in bridgmanite and periclase. Source data

As the creep rate critically depends on the diffusion coefficients, we have re-calculated the creep rates of periclase using another dataset of diffusion coefficients for oxygen in periclase, those of Yoo et al.^40^, which are faster than those of Ita and Cohen^14^ and those of Yang and Flynn^15^. Fitting equation (1) on the data of Yoo et al.^40^ (Extended Data Fig. 1), we determine two new constants for equation (1): *E* = 7200 × 10^−21^ J and ${S}_{0}=2{k}_{{\rm{B}}}$. The activation enthalpies obtained with these new constants are given in Extended Data Table 1 and the evolution of the two sets of diffusion coefficients are compared in Extended Data Fig. 2. The creep rates of periclase with the diffusion coefficients of Yoo et al.^40^ are then slightly faster than with the diffusion coefficients of Ita and Cohen^14^, but still significantly lower than those of bridgmanite (Extended Data Fig. 3). For this comparison, we have also taken into account the uncertainty on the rheology of bridgmanite owing to the lack of constraints on diffusion coefficients (this is discussed in detail by Reali et al.^21^). For this purpose, we vary the concentration of vacancies in bridgmanite *X*_v_ between 10^−3^ and 10^−6^. Extended Data Fig. 2 shows that no matter which combination of data we may choose, Mg (or Si) diffusion in bridgmanite is always significantly faster than oxygen diffusion in periclase. The contrast is particularly marked at high pressures. Extended Data Figs. 3–7 show that even taking into account these uncertainties in the most conservative way does not change any of our conclusions.

#### Influence of iron

Our study is based on the end-member compositions MgO and MgSiO_3_, whereas in the mantle these minerals contain iron (and aluminium for bridgmanite). These differences in composition are likely to affect the values of the diffusion coefficients on which our calculations are based. In bridgmanite, this is taken into account by the range of vacancy concentrations considered^21^, see discussion above and Extended Data Figs. 3, 4, 6 and 7. In ferropericlase, it has been shown that oxygen diffusion coefficients are unaffected by the presence of trivalent cations^13,41^, because these do not alter the concentration of neutral cation–anion vacancy pairs that are responsible for oxygen transport. The addition of significant amounts of FeO, however, is likely to alter oxygen diffusion coefficients. This effect was considered by Reali et al.^42^, who showed that anion diffusion in MgO and in other oxides and halides with rock salt structures is well described by a homologous temperature scaling, wherein the diffusion coefficients as a function of temperature collapse onto a common curve when normalized to the melting temperature. The addition of FeO lowers the melting temperature of periclase, and hence is expected to enhance oxygen diffusivity. We have thus calculated oxygen diffusion coefficients under lower mantle conditions for ferropericlase as a function of its Fe content, under the same assumptions made by Reali et al.^42^. Considering the addition of up to 20 mol% FeO in periclase^43^, we do find that the enhancement of oxygen diffusion is up by a factor of approximately 2000 relative to pure MgO. However, this enhancement does not change the conclusions of this study for the bulk lower mantle, as oxygen diffusion in ferropericlase remains significantly slower than Mg or Si in bridgmanite.

#### Influence of periclase volume fraction

We have computed the rheology of bridgmanite–periclase aggregates for various volume fractions of periclase based on four homogenization procedures (Extended Data Figs. 6 and 7), the difference between them being related to the knowledge on the specimen microstructure that is taken into account.

First, Reuss and Voigt bounds are valid for any microstructures. They only depend on the volume fraction of both phases, not on their geometrical arrangement. They provide rigorous lower and upper bounds for ${\widetilde{\sigma }}_{0}$, respectively. In other words, ${\widetilde{\sigma }}_{0}$ in the aggregate cannot be smaller than ${{\widetilde{\sigma }}_{0}}^{{\rm{Reuss}}}$ nor larger than ${{\widetilde{\sigma }}_{0}}^{{\rm{Voigt}}}$. The Reuss bound is obtained when assuming that both phases are submitted to the same stress, whereas the Voigt bound is obtained when assuming a uniform strain rate in both phases.

The Hashin-Strikman variational upper bound^29^, denoted HS+ in Extended Data Figs. 6 and 7, is another rigorous upper bound for ${\widetilde{\sigma }}_{0}$. It is more stringent than the Voigt upper bound as is based on an extra assumption concerning the specimen microstructure, that is, that the microstructure is isotropic. It is obtained when assuming that the softer phase (here, bridgmanite) is spread as inclusions in a stiffer matrix (periclase) while the associated nonlinear homogenization problem is addressed using the variational procedure introduced by Ponte Castaneda, also known as ‘modified secant’ method^44^.

To stick more closely to the possible rheology of an assemblage representative of the lower mantle, one can reasonably assume that bridgmanite and periclase phases are randomly mixed, that is, that contrary to what is assumed within the HS+ bound, both phases are on the same footing, with neither of them playing the role of a matrix and the other one being inclusions. In that case, a good estimation of the effective behaviour is provided by the POSO extension of the Self-Consistent scheme (SC in Extended Data Figs. 6 and 7), introduced in the previous section. Here, unlike previous applications to polycrystalline aggregates, where individual crystal orientations were taken into account^45,46^, a two-phase aggregate was considered. A fully optimized linearization procedure for the SC scheme has been proposed recently^47,48^ and applied to minerals of the mantle transition zone^49^; results are close to the POSO version when the mechanical contrast between the phases and the nonlinearity of their behaviour is not loo large, as here.

Typical results are shown in Extended Data Figs. 6 and 7, calculated here for pressures between 30 and 120 GPa and X_v_ = 10^−3^–10^−6^. Due to the large mechanical contrast between periclase and bridgmanite, the effective rheology of the aggregate, predicted by the POSO-SC model, lies very close to the Reuss lower bound for volume fractions lower than 30%, whereas it is close to the Voigt upper bound for a volume fraction larger than 70%. In between, the effective stress ${\widetilde{\sigma }}_{0}$ evolves significantly, by more than one order of magnitude. This would correspond to a change in viscosity of more than three orders of magnitude, the viscosity being proportional to ${\left({\widetilde{\sigma }}_{0}\right)}^{n}$. One other specific feature of these results is illustrated in the right panels of Extended Data Figs. 6 and 7, which show the mean equivalent strain rate in the two phases within the aggregate, normalized by the effective strain rate, as predicted by the SC model. It is obtained that the periclase strain rate is really small at volume fractions lower than 40%. This volume fraction corresponds to a ‘mechanical percolation threshold’ observed in previous work^50,51^. At small volume fractions of the highly viscous periclase phase, the aggregate can deform with almost no plastic strain in that phase.

## Online content

Any methods, additional references, Nature Portfolio reporting summaries, source data, extended data, supplementary information, acknowledgements, peer review information; details of author contributions and competing interests; and statements of data and code availability are available at 10.1038/s41586-022-05410-9.

### Source data

Source Data Fig. 1

Source Data Fig. 2

Source Data Fig. 3

Source Data Fig. 4

Source Data Extended Data Fig. 1

Source Data Extended Data Fig. 2

Source Data Extended Data Fig. 3

Source Data Extended Data Fig. 4

Source Data Extended Data Fig. 5

Source Data Extended Data Fig. 6

Source Data Extended Data Fig. 7

## Figures and Tables

**Extended Data Table 1:**
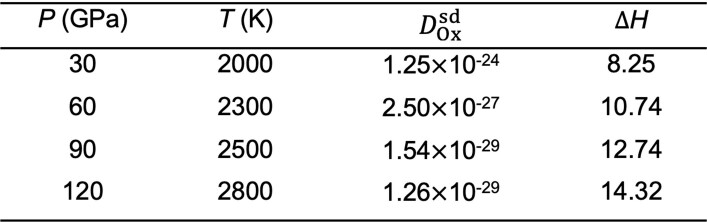
Additional diffusion coefficients

Diffusion coefficients (m²/s) from eq. 1 fit on Yoo et al.^40^ and activation enthalpy (eV) for MgO obtained from eq. 2.

**Extended Data Table 2:**
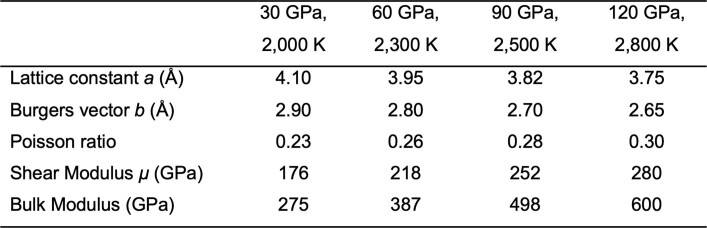
Periclase parameters

Lattice parameters and elastic properties of periclase used in 2.5D Dislocation Dynamics.

## Data Availability

The data of this manuscript are available at 10.5281/zenodo.5970733. Source data are provided with this paper.

## Code availability

The source code of the 2.5D DD is available at 10.5281/zenodo.6306546. 
